# Ferritin Blocks Inhibitory Effects of Two-Chain High Molecular Weight Kininogen (HKa) on Adhesion and Survival Signaling in Endothelial Cells

**DOI:** 10.1371/journal.pone.0040030

**Published:** 2012-07-02

**Authors:** Lia Tesfay, Annissa J. Huhn, Heather Hatcher, Frank M. Torti, Suzy V. Torti

**Affiliations:** 1 Department of Cancer Biology, Wake Forest School of Medicine, Winston-Salem, North Carolina, United States of America; 2 Department of Biochemistry, Wake Forest School of Medicine, Winston-Salem, North Carolina, United States of America; 3 Comprehensive Cancer Center, Wake Forest School of Medicine, Winston-Salem, North Carolina, United States of America; Wayne State University School of Medicine, United States of America

## Abstract

Angiogenesis is tightly regulated through complex crosstalk between pro- and anti-angiogenic signals. High molecular weight kininogen (HK) is an endogenous protein that is proteolytically cleaved in plasma and on endothelial cell surfaces to HKa, an anti-angiogenic protein. Ferritin binds to HKa and blocks its anti-angiogenic activity. Here, we explore mechanisms underlying the cytoprotective effect of ferritin in endothelial cells exposed to HKa. We observe that ferritin promotes adhesion and survival of HKa-treated cells and restores key survival and adhesion signaling pathways mediated by Erk, Akt, FAK and paxillin. We further elucidate structural motifs of both HKa and ferritin that are required for effects on endothelial cells. We identify an histidine-glycine-lysine (HGK) -rich antiproliferative region within domain 5 of HK as the target of ferritin, and demonstrate that both ferritin subunits of the H and L type regulate HKa activity. We further demonstrate that ferritin reduces binding of HKa to endothelial cells and restores the association of uPAR with *α*5*β*1 integrin. We propose that ferritin blocks the anti-angiogenic activity of HKa by reducing binding of HKa to UPAR and interfering with anti-adhesive and anti-proliferative signaling of HKa.

## Introduction

Angiogenesis, the formation of new blood vessels from pre-existing vessels, is carefully regulated by a complex balance between pro- and anti-angiogenic signaling [1]. Disruption of this balance contributes to a number of pathological conditions. These include the excess angiogenesis seen in diabetic retinopathy [2], as well as the aberrant angiogenesis in the tumor microenvironment, which is believed to contribute to tumor progression and metastasis [3]. Conversely, defective angiogenesis can compromise wound healing [4].

High molecular weight kininogen (HK) is a plasma protein originally identified for its role in the intrinsic pathway of coagulation. HK is comprised of six domains, D1–D6. Endoproteolytic cleavage of HK by kallikrein excises domain 4, yielding two bio-active molecules: bradykinin, contained within domain 4, and HKa, composed of domains 1–3 linked through a disulfide bond to domains 5 and 6 [5]. Bradykinin is a potent nonapeptide hormone with a 30 second half-life [6]. Among its other activities, bradykinin binds to G-coupled receptors and triggers NO release, which promotes angiogenesis. HKa, the other product formed from cleavage of HK, has a longer 9 hour half-life [7], and antagonizes bradykinin's activity by serving as endogenous inhibitor of angiogenesis [8]. Proteins that modulate bradykinin or HKa activity therefore have the potential to exert an important effect on the balance between pro- and anti-angiogenic responses.

Ferritin is a 24 subunit protein best known for its role in intracellular iron storage and detoxification (reviewed in [9]). Ferritin is composed of two subunit types, termed H and L, which share considerable sequence similarity. Twenty-four of these subunits assemble to form the mature ferritin protein. The H subunit contains a ferroxidase activity, important in iron oxidation, whereas the L subunit is important in iron nucleation, iron core formation, and protein stability. The ratio of H to L subunits is determined by tissue type and is also modulated by inflammatory and other stimuli.

In addition to its intracellular localization, ferritin exists in the plasma, where it may serve roles in addition to its classic function in intracellular iron storage. For example, extracellular ferritin has been suggested to serve in iron delivery [10], [11], and to exhibit immunosuppressive functions by affecting the proliferation and function of lymphocytes [10], [12]–[14]. Extracellular ferritin appears to contain primarily L subunits, including a truncated version of ferritin L [15]. Serum ferritin is elevated not only in conditions of iron overload, but in acute and chronic inflammation and cancer. Extracellular ferritin binds to cell surface receptors on mouse [16] and human [17] cells, and has been reported to exert a pro-inflammatory effect on hepatic stellate cells [18].

Our laboratory identified ferritin as a protein that regulates the activity of HKa [19]–[21]. In particular, ferritin blocks the anti-angiogenic effects of HKa on endothelial cells both *in vitro* and *in vivo* [20]. HKa exerts its anti-angiogenic effect by inhibiting endothelial cell migration and proliferation, as well as by inducing apoptosis [20], [22]–[24]. Ferritin antagonizes the ability of HKa to both induce apoptosis and inhibit the migration of cultured endothelial cells [20]. Ferritin is an effective HKa antagonist *in vivo*, blocking the inhibitory effect of HKa on angiogenesis in a tumor xenograft and restoring angiogenesis to control levels [20]. Ferritin also inhibits the production of HKa by inhibiting the proteolytic cleavage of its precursor, HK [19], [21].

The mechanism by which ferritin antagonizes HKa is unknown. Ferritin binds to HKa; however, whether this underlies the ability of ferritin to block the anti-angiogenic effect of HKa on endothelial cells has not been investigated. Effects of ferritin on signaling pathways modulated by HKa are also unknown. In this manuscript, we explore the mechanisms underlying the antagonistic effects of ferritin on HKa activity. We find that ferritin antagonizes HKa-mediated anti-angiogenic signaling at two key steps, blocking inhibitory effects of HKa on both survival and adhesion signaling. The interplay between ferritin and HKa, two endogenous proteins, may be important in determining angiogenic outcome in pathologic as well as physiologic settings.

## Results

### Ferritin-mediated enhancement of endothelial cell survival in the presence of HKa is associated with activation of Paxillin, AKT, and Erk

**Figure 1:**
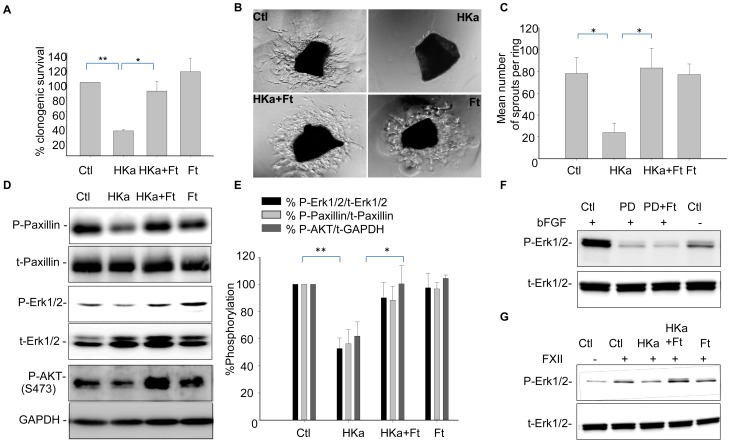
Ferritin restores colony formation, angiogenesis, and phosphorylation of paxillin, Erk1/2, and AKT in HUVECs exposed to HKa. **A.** HUVECs were treated with 50 nM HKa with and without 100 nM ferritin in the presence of 20 ng/ml bFGF for 24 hours. Growth medium was replaced and colonies were allowed to grow for 10 days before fixing and staining with crystal violet. Means and standard deviations of 3 independent experiments are shown with *p<0.01; **p<0.002. **B.** Aortic rings were stimulated with 30 ng/ml VEGF and treated with 100 nM HKa alone or in combination with 200 nM ferritin for 48 hours. Angiogenic sprouts were photographed on day 5. **C.** The number of sprouts were quantified from three different rings for each condition with *p<0.002. **D.** HUVECs were treated with 50 nM HKa alone, 100 nM ferritin alone, or co-treated with HKa and ferritin in basal media containing 20 ng/ml bFGF and 10 µM ZnCl_2_ for 24 hours. Activation of paxillin, Erk and Akt were determined by western blotting using antibodies to phosphorylated (P) and total (T) proteins. **E.** Band intensities were quantified by densitometry using ImageJ. Means and standard deviations of 3 independent experiments are shown, *p<0.02; **p<0.0003. **F.** HUVECs were stimulated as in **D**, and treated with 100 µM PD98059 in presence and absence of 100 nM ferritin. **G.** Cells were stimulated with 62 nM FXII and treated as described in **D**.

We have previously shown that HKa inhibits endothelial cell survival *in vitro* and *in vivo*, and that ferritin antagonizes the antiproliferative activity of HKa [20]. Pro-survival effects of ferritin in HKa-treated cells are dose-dependent (Figure S1), and are observed using either metabolic [20] or clonogenic assays (Figure 1A). *In vivo*, ferritin counteracts the antiangiogenic effects of HKa in the tumor microenvironment [20]. In addition, as shown in Figure 1B, C ferritin blocks the inhibitory effects of HKa in an aortic ring assay, which measures angiogenic sprouting from normal blood vessels *ex vivo* [25]. These results suggest that ferritin may modulate the anti-angiogenic activity of HKa in a broad array of physiological and pathophysiological contexts.

To elucidate the mechanism by which ferritin promotes cell survival and proliferation in endothelial cells exposed to HKa, we assessed effects of ferritin and HKa on survival signaling. In particular, we tested the ability of ferritin to modulate MAPK44/42 (Erk) and AKT, kinases that play central roles in governing cell survival and proliferation in numerous cell types, including endothelial cells [26]–[29]. We also examined effects of ferritin on paxillin, a downstream target of Erk that has been implicated in the apoptotic effects of HKa [30].

Endothelial cells were plated and allowed to adhere. To assess the role of ferritin in modulating the Erk pathway, in some cases cells were stimulated with bFGF or FXII. Cells were then treated with HKa in the presence or absence of ferritin and effects on signaling monitored 24 hours later using antibodies specific to activated Erk, AKT and paxillin. As shown in Figure 1D–E, phosphorylation of Erk, AKT and paxillin were all reduced when endothelial cells were treated with HKa. However, co-treatment with ferritin restored phosphorylation to levels seen in control cells that had not been treated with HKa. Effects of HKa and ferritin were both statistically significant (Figure 1E). Ferritin by itself did not affect any of these pathways (Figure 1). Ferritin did not restore phosphorylation of Erk in cells treated with the Erk inhibitor PD98059 (Fig. 1F), indicating that ferritin does not act as a non-specific stimulator of Erk activity. Similar results were obtained when Erk signaling was induced by treating endothelial cells with Factor XII [31]: HKa reduced phosphorylation of Erk and ferritin reversed this action (Fig. 1G). Collectively, these results demonstrate that ferritin restores Erk and AKT signaling in cells treated with HKa.

### Ferritin promotes endothelial cell adhesion and adhesion signaling in the presence of HKa

We next tested whether ferritin might also contribute to endothelial cell survival by blocking the anti-adhesive activity of HKa, since HKa has been shown to block adhesion of endothelial cells to provisional extracellular matrix proteins (ECM) such as vitronectin [24].

**Figure 2:**
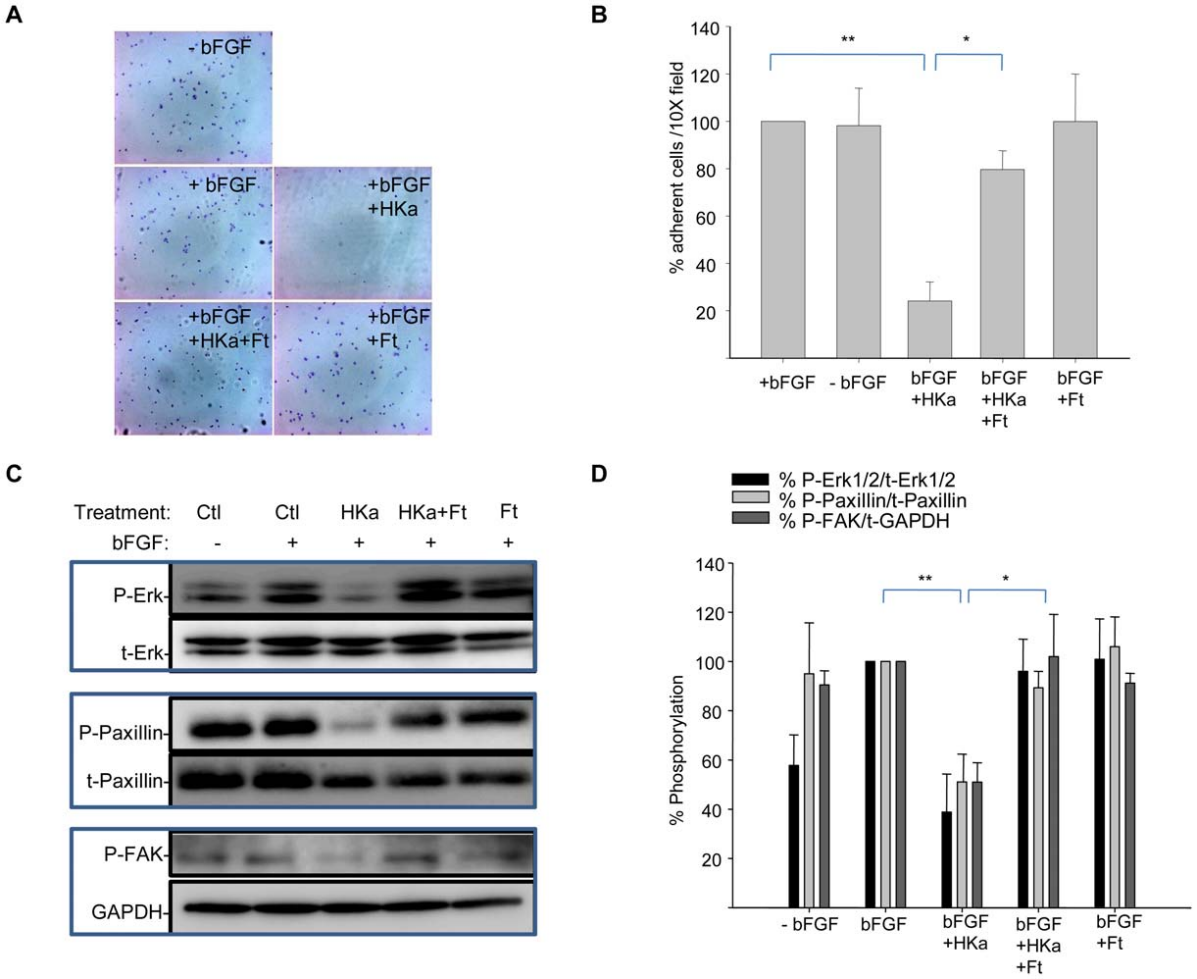
Ferritin reverses the anti-adhesive properties of HKa. Cells were incubated with HKa (50 nM) alone, HKa (50 nM) plus ferritin (100 nM), or ferritin (100 nM) alone and allowed to adhere on vitronectin-coated coverslips in the presence of 20 ng/ml bFGF and 10 µM ZnCl_2_ for two hours. **A.** Adherent cells were fixed and stained with crystal violet and visualized by microscopy; **B.** Adherent cells per 10× field were counted; shown are means and standard deviation of 3 experiments with * p<0.001 and ** p<0.0001. **C.** Adherent cells were lysed and analyzed by western blotting. **D.** Phosphorylation of signaling molecules was quantified by densitometry. Data is expressed as percent phosphorylated/total protein; shown are means and standard deviation of three independent experiments; *p<0.02; **p<0.003.

To test effects of ferritin on adhesion, we first performed a short-term adhesion assay. HUVEC cells were plated on vitronectin-coated dishes in the presence or absence of bFGF. At the time of plating, cells were also treated with HKa alone, ferritin alone, or with the combination of ferritin and HKa. Untreated cells served as a control. Two hours post–plating and treatment, control cells were adherent. Non-adherent cells were removed from the surface by washing, and remaining adherent cells were fixed and stained with crystal violet and visualized by microscopy. As seen in Figure 2A and 2B, HKa significantly inhibited adhesion of cells to vitronectin; however, co-treatment with ferritin enabled the cells to adhere to vitronectin.

Next, we tested whether ferritin-promoted adhesion was associated with restoration of signaling pathways disrupted by HKa. Cell adhesion and spreading are controlled by complex signaling events mediated by proteins of the cell surface and the extracellular matrix. Key kinases in these signaling events are FAK and Erk, which converge on the common downstream target paxillin, an adaptor protein whose phosphorylation is required for integrin-mediated cytoskeletal reorganization [32].

To test effects of ferritin on adhesion signaling, we measured its effect on FAK and Erk signaling under short-term treatment conditions. Endothelial cells were plated in the presence of bFGF with HKa alone, ferritin alone, or the combination of ferritin and HKa on vitronectin-coated plates. Controls were untreated. Cells were lysed two hours later and phosphorylation of FAK, paxillin and Erk were analyzed by western blot. Figure 2C and 2D demonstrates that in the presence of HKa alone, phosphorylation of paxillin and Erk diminished, despite the presence of bFGF. Co-treatment with HKa and ferritin restored phosphorylation of paxillin and Erk. Thus, restoration of short-term adhesion (Figure 2) as well as long-term survival (Figure 1) of HKa-treated endothelial cells by ferritin is associated with reestablishment of critical signaling pathways.

### Ferritin antagonizes the effects of HKa on endothelial cell viability and signaling by binding to an HGK-rich region of HKa

HKa comprises five domains: amino-terminal domains 1–3 linked through a disulfide bond to domains 5 and 6 [33]. Within domain 5 lies a small 29 amino acid region termed the HGK-rich region (histidine-glycine-lysine-rich region from D474 to K502 [20]), that mediates number of key functions of HKa, including binding to the endothelial cell surface [34] and inhibition of adhesion, invasion, and metastasis [35]. We previously observed that ferritin binds to this HGK-rich region of HKa in a cell-free system [21].

**Figure 3:**
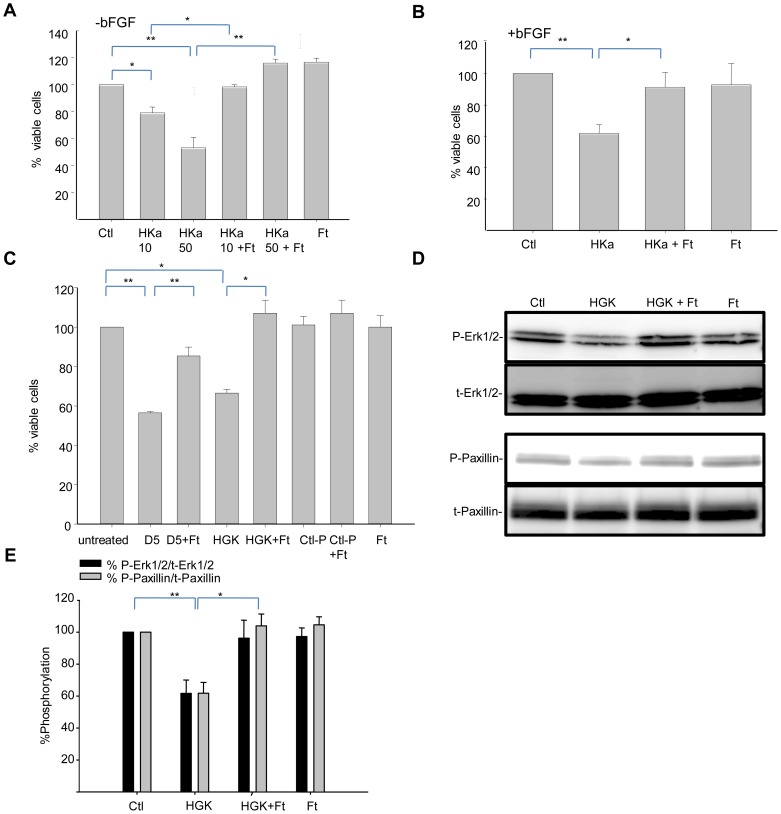
Ferritin modulates HKa-dependent effects on proliferation and adhesion by targeting an HGK-rich region of HKa. **A.** HUVECs were treated with 10 nM or 50 nM HKa and 100 nM ferritin for 24 hours in basal medium containing 10 µM ZnCl_2_ in the absence of growth factors. Viability was assessed using an MTT assay. **B.** Cells were treated as in **A** in the presence of 20 ng/ml bFGF. **C.** HUVEC cells grown in basal medium containing 10 µ M ZnCl_2_ and 20 ng/ml bFGF were treated with 300 nM D5, 2 µM HGK-rich peptide or 2 µM control peptide in the presence or absence of 100 nM ferritin for 24 hours and viability assessed using an MTT assay. Shown are means and standard deviation of triplicate experiments with * p<0.004 and ** p<0.0004. **D.** Cells were allowed to adhere to vitronectin coated dishes for two hours in the presence of 2.5 µM HGK peptide, 100 nM ferritin, or the combination of HGK peptide and ferritin. Phosphorylation of Erk1/2 and paxillin was assessed by western blotting. **F.** Phosphorylation of signaling molecules was quantified by densitometry. Means and standard deviation of 4 independent experiments are shown. *p<0.01; **p<0.002.

We therefore explored whether interaction of ferritin with the HGK-rich domain of HKa is responsible for ferritin's ability to block anti-angiogenic effects of HKa on endothelial cells. We first assessed whether anti-proliferative effects of the HGK-rich D474-K502 domain of HKa could be inhibited by ferritin. Endothelial cells were incubated with HKa or the 29 amino acid HGK-rich domain of HKa in the presence or absence of ferritin. Consistent with previous results [20], HKa inhibited endothelial cell proliferation, and this effect was blocked by ferritin (Figure 3A). Stimulation with growth factors was not able to overcome the inhibitory effect of HKa or modulate ferritin's ability to antagonize HKa (Figure 3B). We then examined if the HGK domain would exert a similar inhibitory effect as HKa on endothelial cells, and if ferritin would block these effects. As shown in Figure 3C, domain 5 of HKa reduced cell viability to 56%±0.3% of control, and co-treatment with ferritin increased cell viability to 85±4.0% of control (p<0.0004). The HGK-rich peptide exerted a similar effect, reducing viability 66%±2.0, whereas a control peptide (G440-L473, corresponding to a 33 amino acid region of HKa located immediately adjacent to the HGK rich region) was not able to affect proliferation (Figure 3C). Co-treatment with ferritin blocked the inhibitory effect of the HGK peptide, but did not affect the response to the control peptide. Thus, ferritin exerts its pro-survival effects on endothelial cells by targeting a small HGK-rich region within domain 5 of HKa.

To test whether the inhibitory effects of ferritin on HKa signaling mapped to the same HGK-rich domain of HKa, we treated cells with the 29 amino acid HGK-rich domain in the presence and absence of ferritin and assessed effects on adhesion signaling. As shown in Figure 3D and 3E, treatment of cells with the HGK rich domain inhibited activation of both Erk and paxillin. Ferritin alone had no effect on activation of these signaling molecules. However, co-treatment with ferritin blocked the inhibitory effect of the HGK rich domain on both phosphorylation of paxillin and Erk. These results demonstrate that ferritin restores adhesion signaling by interfering with the activity of the HGK rich domain of HKa.

### Recombinant human Ferritin-H and Ferritin-L bind to domain 5 of HKa

Ferritin is composed of an admixture of 24 subunits of the H and L type. H and L subunits assemble in various ratios to create “H-rich” (HFt) or “L-rich” (LFt) molecules. The H and L subunits of ferritin exhibit some sequence similarity, but also exhibit functional differences [9] and bind to different receptors [16], [17], [36]. Ferritin found in the plasma (serum ferritin) is composed predominantly of a subunit closely related to the L type [15].

**Figure 4:**
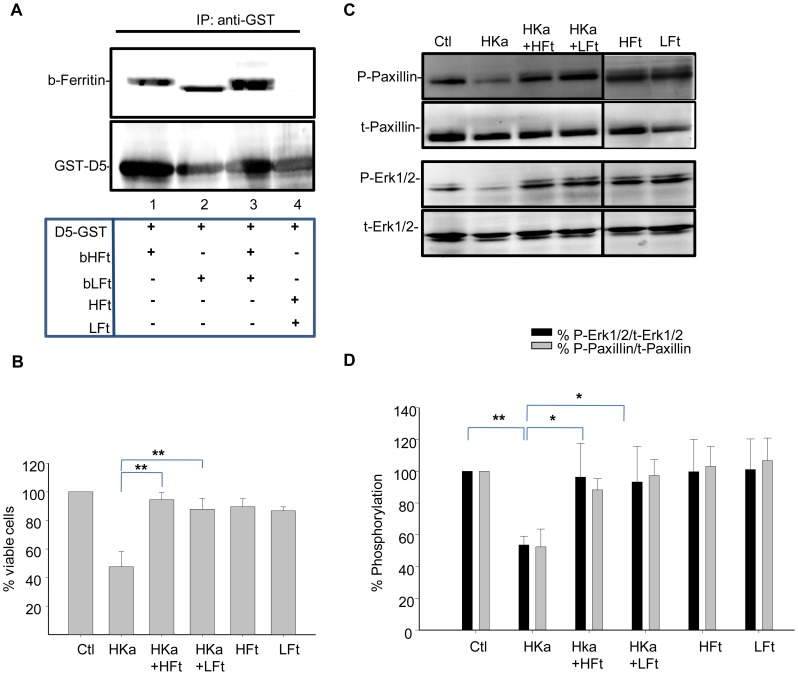
Recombinant HFt and LFt bind to HKa and and inhibit its anti-proliferative activity. **A.** Binding of recombinant HFt and LFt to domain 5 of HKa. Purified and biotinylated recombinant HFt (20 µg) or LFt (20 µg) were incubated with 10 µg GST-D5 and the resulting complexes immunoprecipited with anti-GST antibody. Non-biotinylated HFt and LFt were used in the immunopreciptation shown in lane 4. The membranes were probed with streptavidin-HRP to detect biotinylated ferritin (b-ferritin) as well as with anti-GST antibody. **B.** Cells were treated with 50 nM HKa alone, or co-treated with 100 nM of HFt or LFt in the presence of 20 ng/ml bFGF and 10 µM ZnCl_2_. Cell viability was assessed using an MTT assay 24 hours post-treatment. Shown are means and standard deviation of triplicate experiments with ** p<0.004. **C.** Cell lysates were collected 24 hours post-treatment and analyzed by western blotting. **D.** Phosphorylation of signaling molecules was quantified by densitometry. Shown are means and standard deviation of 3 independent experiments. *p<0.04; **p<0.002. b = biotinylated.

To determine whether ferritin subunit composition affected its ability to antagonize HKa activity, we first tested whether recombinant ferritins bound to HKa in a pull-down experiment. For these experiments we used a protein in which domain 5 of HKa, which contains the HGK-rich peptide sequence, was fused to GST [20]. Recombinant ferritin homopolymers composed either solely of the L subunit or solely of the H subunit [37] were biotinylated, incubated with GST-tagged domain 5 of HKa, and immunoprecipitated with anti-GST antibody. As shown in Figure 4A, ferritins composed of either subunit associated with domain 5 of HKa (lanes 1–4). Controls indicated that GST alone did not associate with either HFt or LFt (Figure S2).

**Figure 5:**
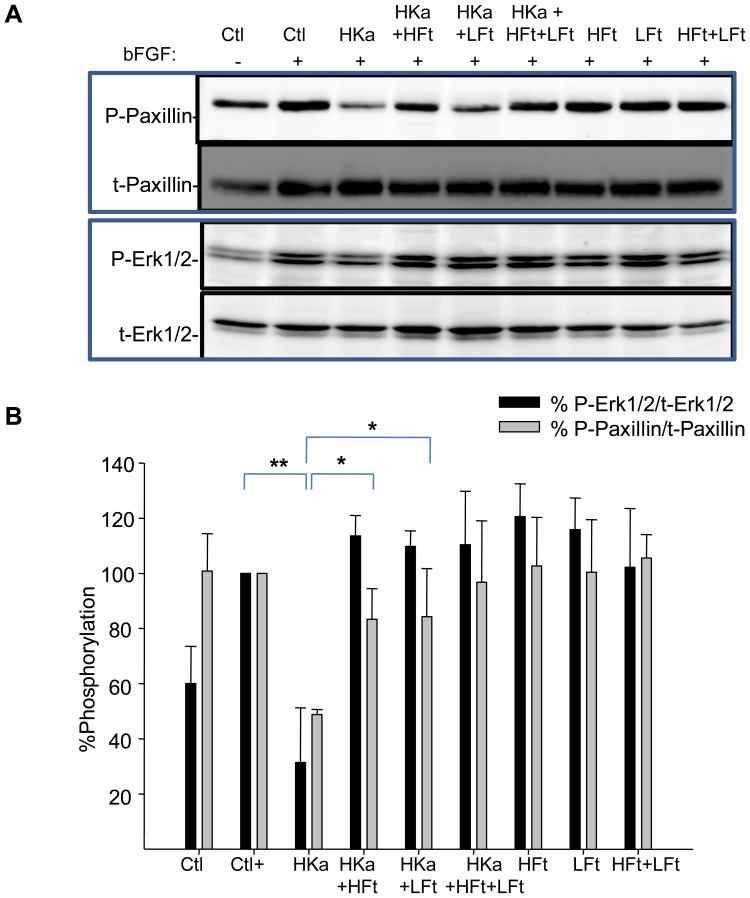
Recombinant HFt and LFt antagonize effects of HKa on adhesion signaling. Cells were suspended in basal medium containing 20 ng/ml bFGF and 10 µ M ZnCl_2_ and allowed to adhere onto vitronectin-coated dishes for 2 hours in the presence of HKa (50 nM), recombinant HFt or LFt(100 nM), or the combination of HKa plus ferritin. **A.** Adherent cells were lysed and analyzed by western blotting. **B.** Phosphorylation was quantified by densitometry; shown are means and standard deviation of 3 independent experiments. *p<0.02; **p<0.004.

We then tested whether HFt and LFt were equally capable of blocking the anti-proliferative effects of HKa on endothelial cells. As shown in Figure 4B, treatment with either HFt or LFt significantly blocked loss of viability in HKa-treated cells (p<0.004). To determine whether these protective effects of HFt and LFt were associated with restoration of adhesion or survival signaling, we treated endothelial cells with HKa in the presence or absence of HFt or LFt or the combination of HFt and LFt for either short or long term, and measured effects on adhesion and survival signaling, respectively. As shown in Figure 4C–D and Figure 5, both recombinant ferritins restored activation of paxillin and Erk in cells treated with HKa. Thus, ferritins composed of either subunit can restore adhesion and survival signaling in endothelial cells treated with HKa.

### Ferritin reduces binding of HKa to endothelial cells and restores association of uPAR with α5β1 integrin

**Figure 6:**
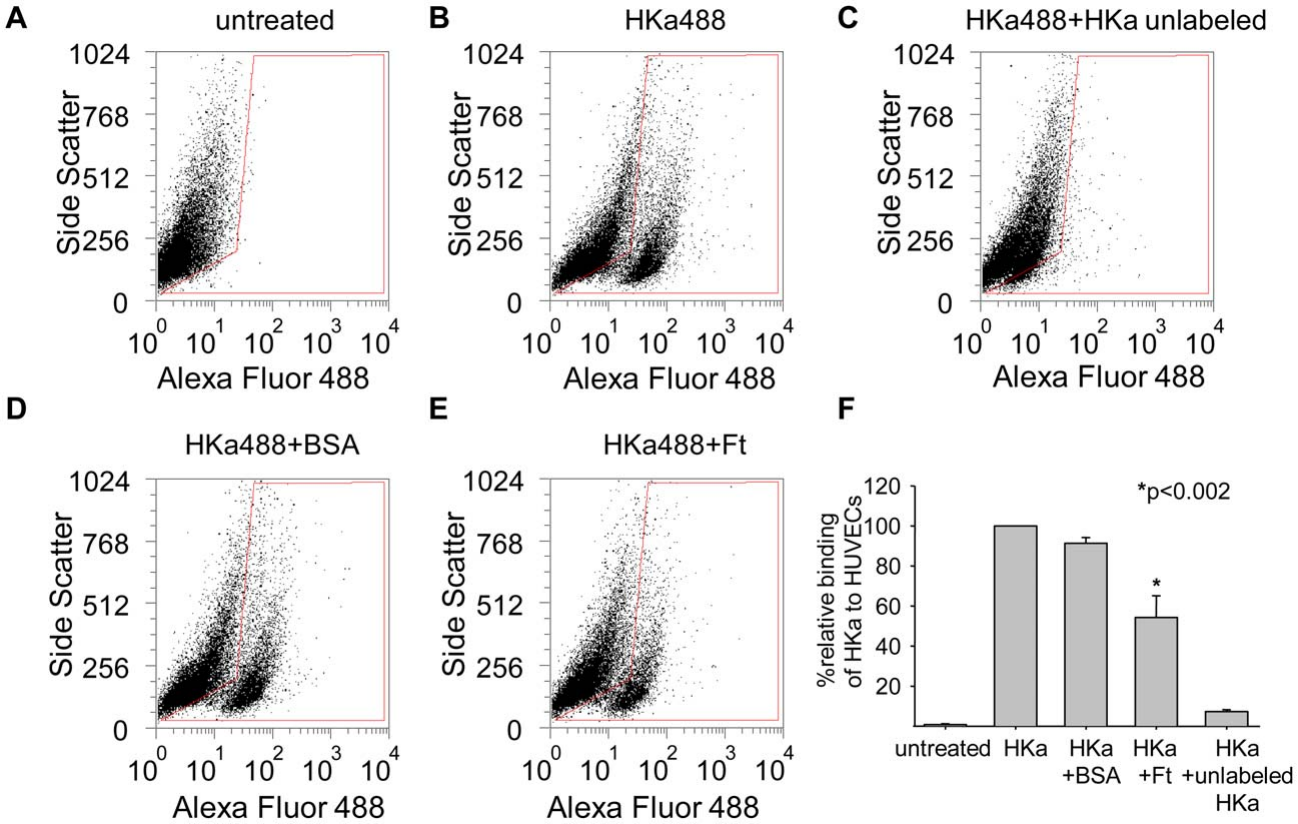
Ferritin inhibits binding of HKa to HUVECs. **A–E.** HUVEC cells were treated with 50 nM Alexa-Fluor 488 labeled HKa ±100 nM unlabeled spleen ferritin or BSA for one hour at 37°C. Cells were analyzed on FACSCaliber using CellQuest Pro Software. **F.** Means and standard deviation of 3 independent experiments.

HKa initiates signaling pathways in endothelial cells by binding to specific cell surface receptors. The binding site for HKa on endothelial cells includes uPAR [38] and other proteins [39], [40]. uPAR is a GPI-linked cell surface receptor that recruits other membrane proteins, including integrins α5β1, into signaling complexes, permitting the triggering of downstream events, such as activation of Erk and Akt [41]. To test whether ferritin blocks HKa signaling by diminishing binding of HKa to the cell surface, we used flow cytometry. As seen in Figure 6, fluorescently labeled HKa exhibited specific binding to HUVECs that was competed by excess unlabeled HKa. Co-treatment of cells with the combination of HKa and ferritin (molar ratio of 1∶2) reduced binding of HKa approximately 50%. Co-treatment of HKa with the control protein BSA did not affect binding.

**Figure 7:**
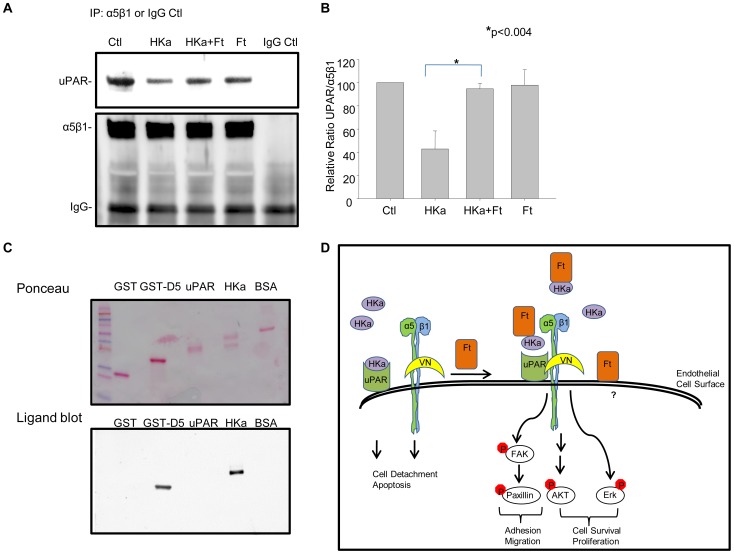
Ferritin blockes HKa-mediated disruption of the complex between uPAR and integrin α5β1. HUVEC cells were allowed to adhere onto vitronectin-coated dishes for 2 hours in the presence of HKa (50 nM), ferritin (100 nM), or the combination of HKa plus ferritin. **A.** Cells were lysed, immunoprecipitated with anti- α5β1 antibody, and the immunoprecipitates analyzed by western blotting with anti-uPAR and anti-β1 antibodies. **B.** Bands were quantified by densitometry. Shown are means and standard deviation of 3 independent experiments. **C.** 2 µg of recombinant proteins were electrophoresed on a SDS-PAGE. Binding to ferritin was analyzed by ligand blotting. **D.** Model of ferritin effects on HKa signaling in endothelial cells.

The binding of HKa to uPAR disrupts uPAR-mediated signaling. We tested whether the diminished binding of HKa to the endothelial cell surface in the presence of ferritin (Figure 6) was sufficient to block the ability of HKa to the uPAR signaling complex. Cells were incubated with either HKa or Ft alone, or with the combination of HKa plus ferritin. Signaling complexes were immunoprecipitated using anti-α5β1 integrin antibody and probed for the presence of uPAR using western blotting. As shown in Figure 7A, B, incubation with HKa dissociated uPAR from α5β1 integrin, consistent with previous reports [38]. However, co-treatment with ferritin restored complex formation between uPAR and integrin α5β1. Thus, incubation with ferritin prevents HKa from disrupting upstream (UPAR-mediated) as well as downstream (Erk and Akt) signaling in endothelial cells. Ligand blotting demonstrates that ferritin binds to HKa but does not directly bind to uPAR (Figure 7C), suggesting that ferritin exerts its effects by displacing HKa from an association with uPAR rather than through direct interaction with uPAR. An overall model depicting effects of ferritin and HKa on endothelial cells is shown in Figure 7D.

## Discussion

In this manuscript, we explore the mechanism underlying the cytoprotective effect of ferritin on endothelial cells exposed to HKa. We observe that (1) ferritin promotes adhesion of HKa-treated cells and restores critical survival and adhesion signaling pathways mediated by Erk, Akt, FAK and paxillin; (2) ferritin inhibits the anti-proliferative and anti-adhesive effects of HKa by targeting the HGK-rich domain of HKa; (3) both ferritins of the H and L subunit type can regulate HKa activity; and (4) ferritin diminishes binding of HKa to endothelial cells and restores the association of uPAR with α5β1 integrin. An overall model depicting effects of ferritin on HKa is shown in Figure 7D.

Ferritin restores multiple signaling pathways disrupted by HKa. These include pathways mediated by Erk and Akt, key pro-survival kinases. Erk signaling is critical to endothelial cell survival and sprouting *in vitro* [26], [27] and *in vivo* [29]; Akt also plays a key role in the endothelial cell response to growth factors [28]. Both of these pathways are targeted by HKa [31], [42]. The ability of ferritin to interfere with HKa-mediated inhibition of these pathways was associated with its ability to promote endothelial cell survival and proliferation (Figure 1, 3). HKa also inhibits endothelial cell adhesion to vitronectin by reducing phosphorylation of FAK and paxillin [24]. We found that in addition to its effects on survival, ferritin blocked inhibitory effects of HKa on adhesion signaling by interfering with HKa-dependent blockade of paxillin and FAK. Ferritin alone exerted no measurable effects on these pathways (Figures 1, 2, 3, 4). These observations may provide a molecular explanation for our findings that ferritin inhibits the antiproliferative and apoptotic effects of HKa on endothelial cells and blocks the anti-angiogenic effect of HKa in an aortic ring angiogenesis assay (Figure 1) and the tumor environment [20].

We further identify uPAR as a key upstream node of ferritin and HKa interaction. Erk, Akt, FAK and paxillin are all downstream of uPAR, suggesting that interference with binding of HKa to uPAR and restoration of uPAR signaling are the proximate events in ferritin-mediated inhibition of HKa activity, as depicted in the model shown in Figure 7D. Supporting this interpretation, we observed that ferritin interacts directly with HKa (Figure 4) but not uPAR (Figure 7), and that ferritin reduces overall binding of HKa to cells (Figure 6). Critically, ferritin restored the association of integrin *α*5*β*1 with uPAR in cells treated with HKa (Figure 7A, B), an event that was associated with activation of Erk, paxillin and FAK (Figure 2). We suggest that collectively, these events act in concert to restore adhesion (Figure 2) and proliferation (Figure 3) in endothelial cells challenged with HKa.

We demonstrate that the HGK-rich domain of HKa is critical to the ability of ferritin to modulate the biological activities of HKa. We previously showed in plate-binding assays that the binding of HKa to ferritin is mediated through a small HGK-rich region of HKa that maps to domain 5 [20]. Supporting previous observations [23], this domain was able to inhibit endothelial cell proliferation and adhesion signaling (Figure 3). We further observed that ferritin was able to counteract effects mediated by this HGK-rich peptide as well as the intact HKa protein (Figure 3).

Binding of ferritin to HKa is not ferritin subunit-specific. We observed that domain 5 of HKa interacted with ferritins composed of either the H or the L ferritin subunit types (Figure 4). Further, both HFt and LFt were able to counteract inhibitory effects of HKa on survival (Figure 4) and adhesive signaling (Figure 5). Although subunit composition varies by tissue type, natural ferritins generally contain some mixture of ferritin H and L subunits. These results suggest that all ferritins, independent of tissue source, are capable of exerting an inhibitory effect on HKa signaling. Human ferritin H and ferritin L have 56% amino acid sequence identity and similar overall structures, both in the monomeric and icositetrahedron form [43], [44]. The ability of both ferritin H and L ferritin subunit types to interact with HKa suggests that binding of HKa to ferritin is mediated by a structural motif or sequence that is shared by the ferritin H and L subunits.

The physiological source of ferritin that binds to HKa is not known. Circulating plasma ferritin is one possibility, and levels of this protein are roughly comparable to endogenous levels of HKa [21]. Because ferritin increases in inflammation or malignancy, plasma ferritin might be particularly effective at inhibiting HKa activity during these conditions. Further, because HKa is produced following docking and cleavage of HK on endothelial cells, locally produced HKa may recruit ferritin from the circulation to the endothelial cell surface. Alternatively or additionally, macrophages, which have recently been identified as a source of secreted ferritin [15], could serve as a local source of ferritin; concentrations of ferritin immediately adjacent to such cells may be appreciable, enabling ferritin to serve as an endogenous regulator of HKa that facilitates maintenance of endothelial cells.

The ability of ferritin to bind to and inhibit the activity of HKa is a non-canonical ferritin function. Ferritin is principally known for its role in iron storage [9]. However, our previous work indicated that holoferritin (containing an iron core) and apoferritin (without iron) exhibited similar capacities to inhibit endothelial cell proliferation [20]. Our current observation that the ferritin subunits, which differ dramatically in their ferroxidase activity, are both able to bind to and inhibit HKa activity further suggests that the ability of ferritin to interact with HKa is independent of its iron storage function. This interpretation is congruent with the evolving view that ferritin may have extracellular signaling activities [18] in addition to its classical role in iron storage.

## Materials and Methods

### Cell culture

Human Umbilical Vein Endothelial Cells (HUVECs) were purchased from Lonza (Walkersville, MD) and cultured in EGM-2 growth medium supplemented with 2% fetal bovine serum at 37°C in a humidified incubator at 5% CO_2_. Cells were used at passages 3–8.

### Cell treatment and viability assays

Cell viability was assayed and quantified using 3-(4,5-Dimethylthiazol-2-yl)-2,5-diphenyltetrazolium bromide (MTT) reagent from Sigma. Cells were seeded in growth medium at a density of 6,000 cells per/well in a 96-well plate, allowed to adhere for 4 hours and the medium was replaced with M199 (Gibco) basal medium without growth factors overnight. Cells were treated in the presence or absence of 20 ng/ml of basic fibroblast growth factor (bFGF; BD Biosciences) and 10 µM ZnCl_2_ in M199 basal starvation medium (GIBCO) with the following reagents: two chain high molecular weight kininogen (HKa; Enzyme Research Laboratories); HGK-rich peptide ^474^DHGHKHKHGHGHGKHKNKGKKNGKHNGWK^502^(Anaspec); control peptide ^440^GHGLGHGHEQQHGLGHEHKFKLDDDLEHQGGHVL^473^(Anaspec); spleen ferritin (Scripps Laboratories), recombinant HFt or LFt (below) for 24 hours prior to MTT assay. For clonogenic assays, colonies were allowed to grow for 10 days before fixing and staining with crystal violet.

### Effect of HKa on signaling molecules in HUVECs

Cells were seeded at 4×10^5^ cells per/well in a 6- well plate in growth medium and allowed to adhere for 4 hours before the medium was replaced with M199 basal medium without growth factors. Following overnight incubation, the cells were either left untreated or treated with HKa, HGK-rich peptide, control peptide or 100 µM PD98059 (Promega) in the presence or absence of ferritin in medium containing 20 ng/ml of bFGF or 62 nM FXII (Haematologic Technologies Inc) and 10 µM ZnCl_2_ for 24 hours. Cells were lysed in Triton X-100 (TX-100) lysis buffer (50 mM Tris, pH 7.5, 150 mM sodium chloride, 0.5% TX-100) supplemented with protease and phosphatase inhibitor cocktails (Roche) and protein concentration of the clarified samples determined using BCA protein assay kit (Pierce). Proteins were separated by SDS PAGE and transferred into a polyvinylidene difluoride (PDVF) membrane. The membranes were blocked in 5% BSA in Tris-buffered-saline containing 0.05% Tween-20, and probed with the following antibodies: anti-phospho-MAPK 44/42, anti-total-MAPK 44/42, anti-phospho-Paxillin Y118, anti-Paxillin, anti- phospho-FAK Y397, anti-FAK, anti-phospho-Akt (Akt S473) (Cell Signaling); anti-GAPDH (Fitzgerald Industries International, Inc.); Streptavidin-HRP (Pierce); and anti-GST (Sigma). Secondary antibodies were either horseradish peroxidase (HRP)-conjugated goat anti-rabbit immunoglobulin G (IgG) or HRP-conjugated goat anti-mouse IgG (Biorad). Signals were detected with SuperSignal West Pico Chemiluminescent Substrate (Thermo Scientific) and membranes were developed with the Luminescent Image Analyzer LAS-3000 (Fujifilm).

### Aortic sprouting

Isolation and preparation of aortic rings were performed essentially as described [25]. In brief, aortas were isolated from 8–12 – week old C57BL/6 mice, transferred into 5 ml Opti-MEM (GIBCO), carefully cleaned of the protective fatty layer and all accessory vessels, and sectioned into 0.5 mm rings. The rings were incubated at 37°C for 2 hours in medium, embedded into growth factor reduced Matrigel (BD), and treated with HKa (100 nM) in presence or absence of spleen ferritin (200 nM) in Opti-MEM supplemented with 2.5% FBS and Penicillin/Streptomycin (GIBCO) plus 30 ng/ml recombinant mouse VEGF (R&D Systems) for 48 hours. Media was replaced and subsequently changed every two days. Aortic rings were photographed at day 5 post treatment. Quantification was performed by counting the number of sprouts originating from the aortic rings.

### Cell adhesion assay

Six-well cell culture plates were coated with 2 ng/ml vitronectin (Promega), blocked with 0.1% bovine serum albumin (BSA) and washed with phosphate buffered saline (PBS). HUVECs (4×10^5^cells/well) were plated in medium containing 20 ng/ml bFGF and 10 µM of ZnCl_2_ and treated with either HKa or HGK-rich peptide in the presence or absence of ferritin. When control cells were adherent (1 to 2 hours at 37°C), all samples were lysed in TX-100 lysis buffer and subjected to Western blotting. To count adherent cells, coverslips were washed with PBS, fixed in 10∶10∶80 acetic acid∶methanol∶water, and treated with 0.4% crystal violet.

### Expression and purification of recombinant HFt and LFt

cDNA encoding the human ferritin H subunit was cloned into the pET-17 bacterial expression vector (Novagen) and transformed into E.coli BL21(DE3) for expression. Following overnight induction with 1 mM IPTG, cell pellets were collected and resuspended in 20 mM Tris-HCl pH 7.5, 1 mM EDTA, 1 mM PMSF, and 10% sucrose. Cells were lysed using an Emulsiflex C-5 cell homogenizer, and cell debris pelleted. The supernatant was heated at 70°C for 10 min, clarified by centrifugation, and passed over a Q-sepharose (Amersham) column equilibrated with 50 mM Tris-HCl pH 7.5. The column was eluted with a gradient of 20 mM-2 M NaCl and the fractions containing HFt were pooled and loaded onto a Superdex S-200 (GE Healthcare) gel filtration column equilibrated with 50 mM Tris-HCl pH 7.5, 200 mM NaCl. Fractions containing HFt were pooled, analyzed for purity via SDS-PAGE electrophoresis, and concentrated to 10 mg/mL. LFt cDNA was similarly cloned into the pET-17 vector and expressed and purified using the same protocol. Endotoxin was removed using Detoxi-gel endotoxin removing gel (Thermo Scientific) and quantified using QCL-1000 Quantitative Chromogenic LAL Assay (Lonza).

### Expression and purification of recombinant HK5

cDNA encoding domain 5 of the human high molecular weight kininogen protein (HK5) was subcloned into the pGEX-6P-1 bacterial expression vector (GE Healthcare) in frame with glutathione S-transferase (GST) and recombinant protein was expressed and purified as described [20].

### Biotinylation of ferritin H and ferritin L

Biotinylation of recombinant HFt and LFt was performed using EZ-Link NHS-PEO4-Biotinylation Kit (Pierce) according to the manufacturer's recommendations. The HABA/Avidin assay (Biotin Quantification Kit, Pierce) was used to estimate molar biotin incorporation.

### GST-pull-down of recombinant proteins

Protein G Sepharose beads (GE Healthcare Biosciences) were washed and incubated with either GST-D5 plus biotinylated HFt, GST-D5 plus biotinylated LFt, GST-D5 plus the combination of biotinylated HFt and LFt, or GST-D5 plus non-biotinylated HFt or LFt at 4°C overnight. Anti-GST antibody (Sigma) was added and incubation continued for an additional 2 hr at 4°C. Beads were washed, centrifuged, and bound complexes analyzed by western blotting.

### Alexa Fluor 488

One mg of HKa or D5 was labeled with Alexa Fluor 488 according to the manufacturer's protocol (Molecular Probes/Invitrogen). Samples were incubated for 1 hour at room temperature and labeled proteins were purified using the purification resin provided with the kit.

### Flow cytometric analysis

Five ×10^5^ HUVEC cells were treated with 50 nM Alexa-Fluor 488-labeled HKa in the presence or absence of 100 nM unlabeled ferritin, 100 nM BSA, or 50-fold excess unlabeled HKa in basal medium containing 20 ng/ml bFGF and 10 µM ZnCl_2_ for one hour at 37°C. Cells were treated in suspension, washed with 1% FBS in PBS and analyzed on FACSCaliber using CellQuest Pro Software.

### Co-immuno-precipitation of UPAR and Integrins α5β1

Endothelial cells were plated on 2 ng/ml vitronectin coated plates. When cells were adherent, the culture medium was replaced with M199 basal medium overnight. Cells were either left untreated or treated with HKa in the presence or absence of ferritin in medium containing 20 ng/ml of bFGF and 10 µM ZnCl_2_ for 24 hours. Cells were lysed in TX-100 lysis buffer and protein concentration of clarified samples determined using BCA protein assay kit (Pierce). For immuno-precipitation 500 µg of cell lysates were incubated with an anti-α5β1 antibody (Millipore) at 4°C overnight with agitation. Beads were washed with 1% BSA in lysis buffer, added to samples, and incubated for 2 hours. Bound complexes were centrifuged and subjected to SDS-PAGE. Membranes were probed with anti-UPAR antibody (Santa Cruz) and anti-β1 integrin antibody (Millipore).

### Ligand blot

Ferritin binding to immobilized recombinant proteins was determined by a ligand blot as previously described [21].

### Statistical Analysis

Results were analyzed by one-way ANOVA followed by pairwise comparison using Students' t-test. Data was considered significant at p≤0.05.

## Supporting Information

Figure S1 **Effects of ferritin on HKa-Mediated inhibition of endothelial cell viability are dose-dependent.** HUVECs were treated with nothing (CTL) or 50 nM HKa in the presence of increasing concentrations of Ft (0–400 nM) for 24 hours and viability assessed using an MTT assay. Cells were also treated with various concentrations of ferritin alone. Shown are means and standard deviations of triplicate determinations.
(TIF)

Figure S2 **GST does not bind ferritin non-specifically.** Lanes 1–4: Purified and biotinylated recombinant HFt (20 µg) or LFt (20 µg) were incubated with 10 µg recombinant GST and the resulting complexes immunoprecipited with anti-GST antibody. Non-biotinylated HFt and LFt were used in the immunopreciptation shown in lanes 4. Lanes 5–7:2 µg of biotinylated HFt, LFt and GST were electrophoresed individually. Membranes were probed with streptavidin-HRP to detect biotinylated ferritin (B-ferritin) as well as with anti-GST antibody.
(TIF)

## References

[1] Folkman J (2003). Fundamental concepts of the angiogenic process.. Curr Mol Med.

[2] Rajappa M, Saxena P, Kaur J (2010). Ocular angiogenesis: mechanisms and recent advances in therapy.. Adv Clin Chem.

[3] Kelly RJ, Darnell C, Rixe O (2010). Target inhibition in antiangiogenic therapy a wide spectrum of selectivity and specificity.. Cancer J.

[4] Zins SR, Amare MF, Tadaki DK, Elster EA, Davis TA (2010). Comparative analysis of angiogenic gene expression in normal and impaired wound healing in diabetic mice: effects of extracorporeal shock wave therapy.. Angiogenesis.

[5] Lalmanach G, Naudin C, Lecaille F, Fritz H (2010). Kininogens: More than cysteine protease inhibitors and kinin precursors.. Biochimie.

[6] Cyr M, Lepage Y, Blais C, Gervais N, Cugno M (2001). Bradykinin and des-Arg(9)-bradykinin metabolic pathways and kinetics of activation of human plasma.. Am J Physiol Heart Circ Physiol.

[7] Schmaier AH, Wahl R, Fisher SJ, Brenner D (1998). The pharmacokinetics of the kininogens.. Thromb Res.

[8] Guo YL, Colman RW (2005). Two faces of high-molecular-weight kininogen (HK) in angiogenesis: bradykinin turns it on and cleaved HK (HKa) turns it off.. J Thromb Haemost.

[9] Torti FM, Torti SV (2002). Regulation of ferritin genes and protein.. Blood.

[10] Leimberg MJ, Prus E, Konijn AM, Fibach E (2008). Macrophages function as a ferritin iron source for cultured human erythroid precursors.. J Cell Biochem.

[11] Sibille JC, Kondo H, Aisen P (1988). Interactions between isolated hepatocytes and Kupffer cells in iron metabolism: a possible role for ferritin as an iron carrier protein.. Hepatology.

[12] Gray CP, Franco AV, Arosio P, Hersey P (2001). Immunosuppressive effects of melanoma-derived heavy-chain ferritin are dependent on stimulation of IL-10 production.. Int J Cancer.

[13] Fargion S, Fracanzani AL, Cislaghi V, Levi S, Cappellini MD (1991). Characteristics of the membrane receptor for human H-ferritin.. Curr Stud Hematol Blood Transfus.

[14] Matzner Y, Hershko C, Polliack A, Konijn AM, Izak G (1979). Suppressive effect of ferritin on in vitro lymphocyte function.. Br J Haematol.

[15] Cohen LA, Gutierrez L, Weiss A, Leichtmann-Bardoogo Y, Zhang DL (2010). Serum ferritin is derived primarily from macrophages through a nonclassical secretory pathway.. Blood.

[16] Chen TT, Li L, Chung DH, Allen CD, Torti SV (2005). TIM-2 is expressed on B cells and in liver and kidney and is a receptor for H-ferritin endocytosis.. J Exp Med.

[17] Li L, Fang CJ, Ryan JC, Niemi EC, Lebron JA (2010). Binding and uptake of H-ferritin are mediated by human transferrin receptor-1.. Proc Natl Acad Sci U S A.

[18] Ruddell RG, Hoang-Le D, Barwood JM, Rutherford PS, Piva TJ (2009). Ferritin functions as a proinflammatory cytokine via iron-independent protein kinase C zeta/nuclear factor kappaB-regulated signaling in rat hepatic stellate cells.. Hepatology.

[19] Coffman LG, Brown JC, Johnson DA, Parthasarathy N, D'Agostino RB (2008). Cleavage of high-molecular-weight kininogen by elastase and tryptase is inhibited by ferritin.. Am J Physiol Lung Cell Mol Physiol.

[20] Coffman LG, Parsonage D, D'Agostino R, Torti FM, Torti SV (2009). Regulatory effects of ferritin on angiogenesis.. Proc Natl Acad Sci U S A.

[21] Parthasarathy N, Torti SV, Torti FM (2002). Ferritin binds to light chain of human H-kininogen and inhibits kallikrein-mediated bradykinin release.. Biochem J.

[22] Guo YL, Wang S, Colman RW (2001). Kininostatin, an angiogenic inhibitor, inhibits proliferation and induces apoptosis of human endothelial cells.. Arterioscler Thromb Vasc Biol.

[23] Colman RW, Jameson BA, Lin Y, Johnson D, Mousa SA (2000). Domain 5 of high molecular weight kininogen (kininostatin) down-regulates endothelial cell proliferation and migration and inhibits angiogenesis.. Blood.

[24] Guo YL, Wang S, Cao DJ, Colman RW (2003). Apoptotic effect of cleaved high molecular weight kininogen is regulated by extracellular matrix proteins.. J Cell Biochem.

[25] Baker M, Robinson SD, Lechertier T, Barber PR, Tavora B (2012). Use of the mouse aortic ring assay to study angiogenesis.. Nat Protoc.

[26] Hood JD, Frausto R, Kiosses WB, Schwartz MA, Cheresh DA (2003). Differential alphav integrin-mediated Ras-ERK signaling during two pathways of angiogenesis.. J Cell Biol.

[27] Mavria G, Vercoulen Y, Yeo M, Paterson H, Karasarides M (2006). ERK-MAPK signaling opposes Rho-kinase to promote endothelial cell survival and sprouting during angiogenesis.. Cancer Cell.

[28] Somanath PR, Razorenova OV, Chen J, Byzova TV (2006). Akt1 in endothelial cell and angiogenesis.. Cell Cycle.

[29] Srinivasan R, Zabuawala T, Huang H, Zhang J, Gulati P (2009). Erk1 and Erk2 regulate endothelial cell proliferation and migration during mouse embryonic angiogenesis.. PLoS One.

[30] Colman RW (2006). Regulation of angiogenesis by the kallikrein-kinin system.. Curr Pharm Des.

[31] LaRusch GA, Mahdi F, Shariat-Madar Z, Adams G, Sitrin RG (2010). Factor XII stimulates ERK1/2 and Akt through uPAR, integrins, and the EGFR to initiate angiogenesis.. Blood.

[32] Deakin NO, Turner CE (2008). Paxillin comes of age.. J Cell Sci.

[33] DeLa Cadena RA, Colman RW (1991). Structure and functions of human kininogens.. Trends Pharmacol Sci.

[34] Hasan AA, Cines DB, Herwald H, Schmaier AH, Muller-Esterl W (1995). Mapping the cell binding site on high molecular weight kininogen domain 5.. J Biol Chem.

[35] Kawasaki M, Maeda T, Hanasawa K, Ohkubo I, Tani T (2003). Effect of His-Gly-Lys motif derived from domain 5 of high molecular weight kininogen on suppression of cancer metastasis both in vitro and in vivo.. J Biol Chem.

[36] Li JY, Paragas N, Ned RM, Qiu A, Viltard M (2009). Scara5 is a ferritin receptor mediating non-transferrin iron delivery.. Dev Cell.

[37] Rucker P, Torti FM, Torti SV (1997). Recombinant ferritin: modulation of subunit stoichiometry in bacterial expression systems.. Protein Eng.

[38] Cao DJ, Guo YL, Colman RW (2004). Urokinase-type plasminogen activator receptor is involved in mediating the apoptotic effect of cleaved high molecular weight kininogen in human endothelial cells.. Circ Res.

[39] McCrae KR, Donate F, Merkulov S, Sun D, Qi X (2005). Inhibition of angiogenesis by cleaved high molecular weight kininogen (HKa) and HKa domain 5.. Curr Cancer Drug Targets.

[40] Peerschke EI, Ghebrehiwet B (2007). The contribution of gC1qR/p33 in infection and inflammation.. Immunobiology.

[41] Smith HW, Marshall CJ (2010). Regulation of cell signalling by uPAR.. Nat Rev Mol Cell Biol.

[42] Liu Y, Cao DJ, Sainz IM, Guo YL, Colman RW (2008). The inhibitory effect of HKa in endothelial cell tube formation is mediated by disrupting the uPA-uPAR complex and inhibiting its signaling and internalization.. Am J Physiol Cell Physiol.

[43] Lawson DM, Artymiuk PJ, Yewdall SJ, Smith JM, Livingstone JC (1991). Solving the structure of human H ferritin by genetically engineering intermolecular crystal contacts.. Nature.

[44] Wang Z, Li C, Ellenburg M, Soistman E, Ruble J (2006). Structure of human ferritin L chain.. Acta Crystallogr D Biol Crystallogr.

