# Supplementary figures and images for: Ferritin Blocks Inhibitory Effects of Two-Chain High Molecular Weight Kininogen (HKa) on Adhesion and Survival Signaling in Endothelial Cells
Source: PLoS One. 2012 Jul 2;7(7):e40030. doi: 10.1371/journal.pone.0040030 (PMC3388046; doi:10.1371/journal.pone.0040030)

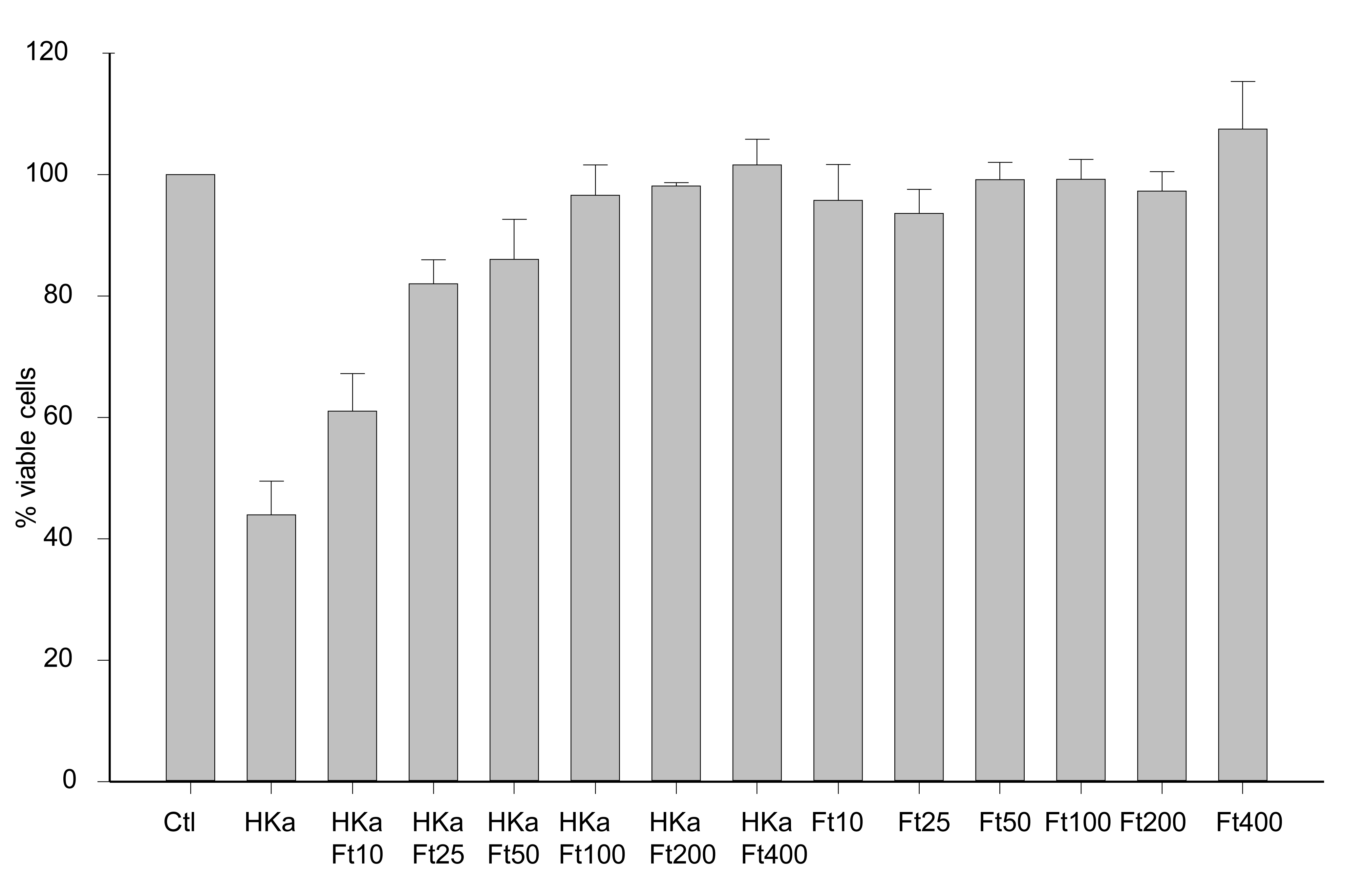

Supplement: Figure S1 — Effects of ferritin on HKa-Mediated inhibition of endothelial cell viability are dose-dependent. HUVECs were treated with nothing (CTL) or 50 nM HKa in the presence of increasing concentrations of Ft (0–400 nM) for 24 hours and viability assessed using an MTT assay. Cells were also treated with various concentrations of ferritin alone. Shown are means and standard deviations of triplicate determinations. (TIF) [file pone.0040030.s001.tif]

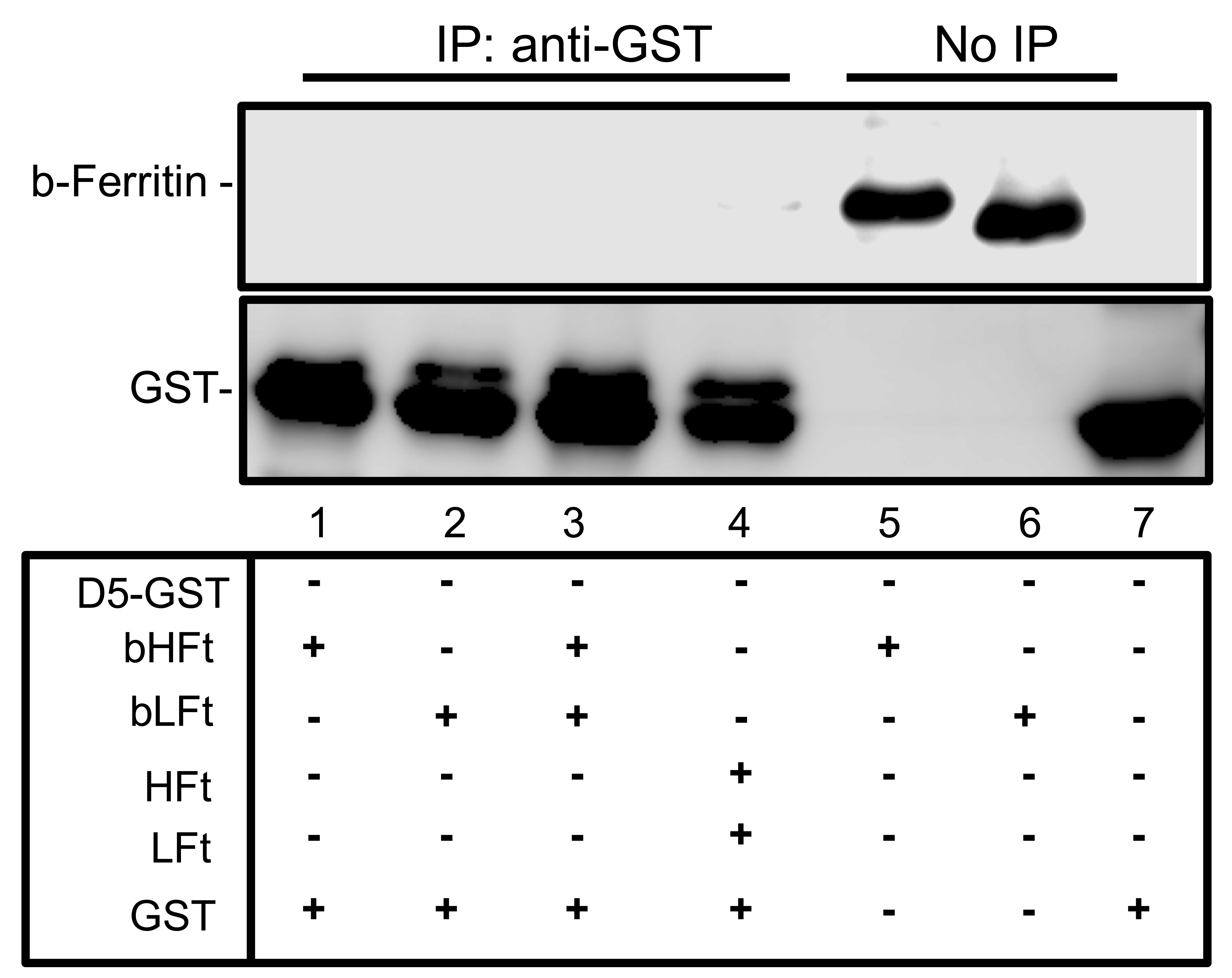

Supplement: Figure S2 — GST does not bind ferritin non-specifically. Lanes 1–4: Purified and biotinylated recombinant HFt (20 µg) or LFt (20 µg) were incubated with 10 µg recombinant GST and the resulting complexes immunoprecipited with anti-GST antibody. Non-biotinylated HFt and LFt were used in the immunopreciptation shown in lanes 4. Lanes 5–7:2 µg of biotinylated HFt, LFt and GST were electrophoresed individually. Membranes were probed with streptavidin-HRP to detect biotinylated ferritin (B-ferritin) as well as with anti-GST antibody. (TIF) [file pone.0040030.s002.tif]
